# Sculpting dynamical systems for models of neural computation and memory

**DOI:** 10.1186/1471-2202-14-S1-P103

**Published:** 2013-07-08

**Authors:** Adam P Trischler, Gabriele MT D'Eleuterio

**Affiliations:** 1 Institute for Aerospace Studies, University of Toronto, Toronto, Ontario, M3H 5T6, Canada

## 

Dynamical systems are now a mainstay of computational neuroscience. Persistent activity in biological neural networks has been posited to result from dynamical attractors in neural state-space [1], and computation with attractors underlies a variety of models for information processing and memory function in the brain [2-4]. Nevertheless, there exists no general, substrate-free method for the construction of these dynamical systems [2], substrate-free meaning not tied to a particular neural instantiation.

We introduce such a method in this work, with a focus on applications to neural computation and memory modeling. We call this method *state-space sculpting*. The systems it constructs, or sculpts, are realized as ordinary differential equations. Sculpting is therefore substrate free; it is general in the sense that sculpted systems may contain any number of attractors embedded at any locations in the state-space and the basins of those attractors may be shaped with any smooth geometry. We conjecture that the sculpting process is analogous to learning by synaptic modification in some biological and artificial neural networks.

The method works as follows. First, a set of attractor locations and types (fixed point, periodic, strange) is defined. Then a set of corresponding vector fields is identified: Each of these has one of the defined attractor types at one of the defined locations in its state-space. Next, the basins of these attractors are delineated by defining smooth boundaries for the corresponding vector fields. Finally, the set of vector fields and boundaries is unified into a single smooth dynamical equation using regularization [5].

We have sculpted various 2-dimensional systems by this method, and their dynamics were confirmed in simulation to exhibit the defined attractors and basins. These systems contained arbitrary combinations of fixed point and periodic attractors, as well as repelling regions, with arbitrary basin geometries. Systems of saddle equilibria were also sculpted to demonstrate the versatility of the method. These can be made to exhibit heteroclinic channels and cycles, which are key features of the Winnerless Competition Principle of neural computation [4].

As a first test of our conjecture on the analogy between sculpting and synaptic modification, the well known Hopfield network [6] was analyzed and characterized in sculpting terms. This analysis demonstrated most notably that the network's basins of attraction are delineated by combinations of codimension-1 hyperplanes containing saddle equilibria. The analogue of state-space sculpting in the Hopfield network is therefore limited, since attractor basins cannot be shaped with any smooth geometry.

Moving forward, it is necessary to study more complicated networks to show that they can be cast in the sculpting framework, as well as to extend the sculpting method to higher-dimensional state-spaces and manifolds.

## References

[B1] Amit DJ Modeling brain function: The world of attractor neural networks 1989 NewYork: Cambridge University Press

[B2] Eliasmith C A unified approach to building and controlling spiking attractor networks Neural Networks 2005 17 6 1276 1314 10.1162/0899766053630332 15901399

[B3] Kaneko K Tsuda I Chaotic Itinerancy Chaos 2003 13 926 936 10.1063/1.1607783 12946185

[B4] Afraimovich VS Rabinovich MI Varona P Heteroclinic contours in neural ensembles and the Winnerless Competition principle International Journal of Bifurcation and Chaos 2004 14 4 1195 1208 10.1142/S0218127404009806

[B5] Sotomayer J Texeira MA Regularization of discontinuous vector fields International Conference on Differential Equations 1995 Lisboa 1195 1208

[B6] Hopfield JJ Neural networks and physical systems with emergent computational capabilities Proc Nat Acad Sc 1982 79 2554 2558 10.1073/pnas.79.8.2554 6953413 PMC346238

